# Negative influence of age and low baseline CD4 on T helper cell recovery among HIV-infected individuals with fixed-dose combination of tenofovir disoproxil, lamivudine and dolutegravir

**DOI:** 10.3389/fpubh.2026.1729238

**Published:** 2026-02-26

**Authors:** Shanmugam Saravanan, Ramachandran Vignesh, Sakthi Rengarajan, Thiyagarajan Vivekanandan, Yean Kong Yong, Venkatesan Varsha, Princy Mounika, Sathasivam Sivamalar, Madhavan Vidya, Esaki M. Shankar, Marie Larsson, Vijayakumar Velu, Sivadoss Raju, Pachamuthu Balakrishnan, Boopathy Nisha, Arcot R. Venkateswaran, Rajendran Kannan

**Affiliations:** 1 Saveetha Medical College and Hospitals (SMCH), Saveetha Institute of Medical and Technical Sciences (SIMATS), Chennai, India; 2 Pre-Clinical Department, Faculty of Medicine, Royal College of Medicine Perak, Universiti Kuala Lumpur, Ipoh, Malaysia; 3 Laboratory Centre, Xiamen University Malaysia, Sepang, Selangor, Malaysia; 4 Saveetha College of Allied Health Sciences, Saveetha Institute of Medical and Technical Sciences (SIMATS), Chennai, India; 5 Department of Research, Meenakshi Academy of Higher Education and Research, Chennai, India; 6 Aevum BioLabs Pvt. Ltd., Hyderabad, India; 7 Infection and Inflammation, Department of Biotechnology, Central University of Tamil Nadu, Thiruvarur, India; 8 Division of Molecular Medicine and Virology, Department of Biomedical and Clinical Sciences, Linköping University, Linköping, Sweden; 9 Division of Microbiology and Immunology, Emory National Primate Research Center, Emory Vaccine Center, Department of Pathology and Laboratory Medicine, Emory University School of Medicine, Atlanta, GA, United States; 10 State Public Health Laboratory, Directorate of Public Health and Preventive Medicine, Chennai, India

**Keywords:** discordant, highly active antiretroviral therapy (HAART), human immunodeficiency virus/acquired immunodeficiency syndrome (HIV/AIDS), immunological discordant, virological discordant

## Abstract

**Background:**

A discordant immune response (DIR) refers to inadequate CD4^+^ T cell recovery despite virological suppression after antiretroviral therapy (ART).

**Methods:**

This study evaluated DIR in HIV-positive patients receiving a first-line regimen of tenofovir disoproxil fumarate, lamivudine and dolutegravir (TLD) at the ART Centre, Saveetha Medical College and Hospital, Chennai, India. DIR was defined as virological suppression to <150 copies/mL and a CD4^+^ T cell count of <500 cells/μL after at least 12 months of ART. Patients with DIR were further categorized as DIR350 (CD4^+^ T-cell count <350 cells/μL) and DIR500 (CD4^+^ T-cell count between 350 and <500 cells/μL). Patients with a CD4^+^ T-cell count >500 cells/μL were classified as responders. Descriptive and ordinal regression analyses were performed, with suitable measures of central trends, and a *p*-value of 0.05 indicating the confirmation of a relationship between the variables.

**Results:**

Out of the 112 patients screened, 40 were enrolled in the study. Of the 40 participants 55% (22/40) showed DIR, with equal proportions in DIR350 (27.5%) and DIR500 (27.5%), while 45% (18/40) achieved immune reconstitution. By using ordinal regression analysis, the study found that the baseline CD4 counts were significantly associated with DIR. Univariate analysis indicated that age, baseline CD4 and treatment duration were significantly associated with CD4 recovery.

**Conclusion:**

The study highlights that, despite viral suppression with TLD, a substantial proportion of patients fail to achieve adequate immune restoration. Lower baseline CD4 was observed to be a key predictor of DIR. These findings underscore the need for early ART initiation, targeted adherence support, and counselling to optimize outcomes and progress toward the UNAIDS 95 target.

## Introduction

Antiretroviral therapy (ART), the cornerstone of HIV management, has transformed the therapeutic landscape for people living with HIV (PLWH) since its debut. By successfully inhibiting HIV viral replication, ART significantly boosts immune function and lowers the morbidity associated with AIDS (1). Immune reconstitution in PLWH receiving ART is generally described as a triphasic process. The first phase, occurring within 1–6 months of ART initiation, is characterized by a rapid increase in CD4^+^ T-cell counts of approximately 20–30 cells/μL per month, primarily due to redistribution of memory CD4^+^ T cells from lymphoid tissues into peripheral circulation. The second phase, extending from 6 months to approximately 2 years, shows a slower rise of 5–10 cells/μL per month and is largely driven by thymic output and *de novo* T-cell production. The third phase, which may persist for up to 7 years or longer, is marked by a gradual increase of 2–5 cells/μL per month (2, 3).

Although the kinetics of immune reconstitution are well described, there still remains a debate on the threshold of CD4 T cell count that defines optimal immunological recovery in PLWH under ART (4). Several studies have demonstrated that individuals who achieve a CD4^+^ T-cell counts exceeding 500 cells/μL while on ART, have morbidity and mortality rates similar to those of HIV-negative individuals, along with reduced risk of non-AIDS related events and malignancies, and are therefore considered to have achieved immunocompetence (2–7). On the other hand, individuals with CD4^+^ T-cell counts below 200 cells/μL or between 200–350 cells/μL are reported to experience significantly higher morbidity from AIDS-defining events and non-AIDS-defining illnesses compared with those whose CD4^+^ T-cell counts exceed 500 cells/μL (8). Consequently, a CD4^+^ T-cell threshold of >500 cells/μL has gained widespread acceptance as an indicator of satisfactory immune reconstitution in PLWH on ART (5), whereas failure to achieve this level of recovery has been associated with poorer clinical outcomes and adverse disease prognosis (9).

The efficacy of ART is traditionally evaluated using two parameters: immunological recovery, namely the increase in CD4^+^ T-cell count (CD4), and the viral suppression to an undetectable level. However, approximately 12–23% of individuals on ART fail to achieve concordant improvement in both parameters and demonstrate a response in only one of these domains, a phenomenon referred to as discordant response (10, 11). When this discordance is characterized by inadequate immunological reconstitution despite sustained virological suppression, it is termed as low CD4^+^ T-cell recovery or discordant immune response (DIR) (12), and represents a significant challenge in the long-term management of HIV infection. DIR is typically characterized by suboptimal CD4^+^ T-cell recovery—often defined as failure to reach CD4^+^ T-cell counts above 500 cells/μL—despite effective viral suppression (12). Individuals exhibiting this pattern of discordance are also described as immunological non-responders (INRs) and have been defined in the past based on CD4 count relative or absolute recovery. In studies that use relative CD4 count recovery from baseline, thresholds varied from less than 20 to 25% or 30% (13–15). Other studies consider the achievement of a predefined CD4 value, with thresholds that range from 200 to 500 cells/μL (16–18).

Owing to heterogeneity in study populations and variability in definitions, the reported prevalence of discordant immune response (DIR) ranges widely from 10 to 40% (13, 19). Multiple factors have been implicated in the development of DIR, including low baseline CD4^+^ T-cell counts, delayed initiation of ART, co-infections, and inter-individual variability in immune recovery. Persistently low CD4^+^ T-cell counts despite viral suppression have been consistently associated with an increased risk of clinical events and mortality (20, 21). Early damage to the immune system prior to ART initiation—through direct HIV-mediated effects on thymic tissue, depletion of hematopoietic progenitor cells, impaired thymic output, and lymphoid tissue fibrosis—may contribute to incomplete immune reconstitution, particularly among individuals initiating ART at advanced stages of disease (22–26). Furthermore, the degree of immune activation at the time of ART initiation has been shown to influence the development of DIR (20) and to independently predict mortality among individuals receiving ART (27).

India is home to over 2 million people living with HIV, this problem should be addressed in order to accomplish the ambitious 95–95–95 plan. The World Health Organization (WHO) and UNAIDS have outlined the 95–95–95 targets as a global strategy to end the HIV epidemic by 2030, aiming for 95% of people living with HIV to know their HIV status, 95% of those diagnosed to receive sustained ART, and 95% of those on ART to achieve virological suppression (22). While these targets primarily focus on diagnosis, treatment coverage, and virological outcomes, they do not explicitly account for immunological recovery, and persistent DIR may indirectly compromise the long-term clinical impact and sustainability of these goals. Although the overall prevalence of DIR remains relatively low in the ART era, its associated comorbidities pose a significant public health challenge, particularly in low and middle income countries. While most research has focused on CD4 reconstitution following ART, the mechanisms underlying DIR, remain poorly understood. The current HIV treatment guidelines offer limited guidance on the clinical management of virologically suppressed patients with inadequate immune recovery.

In this context, the present exploratory cross-sectional study aims to estimate the proportion of PLWH receiving antiretroviral therapy with discordant immune response (DIR) and to identify the clinical and immunological factors associated with this condition.

## Materials and methods

**Figure 1:**
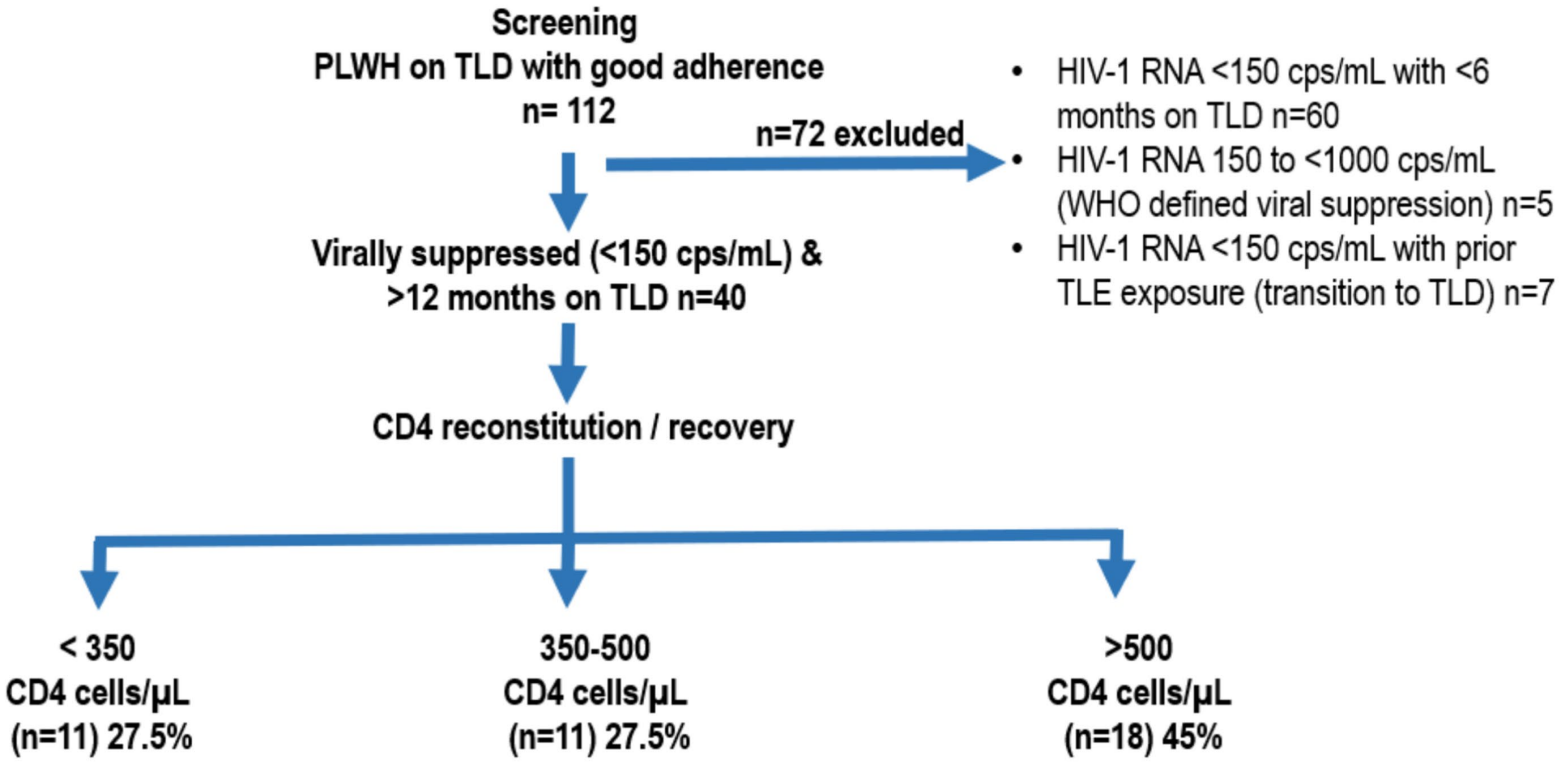
Flowchart describing patient screening and enrollment. PLWH, people living with HIV; TLD, tenofovir disoproxil fumarate (TDF), lamivudine (3TC), and dolutegravir; WHO, World Health Organization; TLE, tenofovir/lamivudine/efavirenz; PVL, plasma viral load.

This exploratory cross-sectional study enrolled HIV-1-positive patients attending the Saveetha Medical College and Hospital ART Centre (SMCH ART Centre) in southern India for their routine care and follow-up visits as per NACO between June 2023 and December 2024. The inclusion criteria comprised of HIV-1 positive patients above 18 years of age who had been receiving ART treatment containing tenofovir, lamivudine and dolutegravir (TLD) for at least one year, had baseline CD4 data recorded in the database and provided informed consent. Figure 1 represents the flow chart of study population.

Blood samples were collected from the patients at the time of enrollment and tested for PVL with Abbott m2000rt real-time PCR (Abbott Molecular Inc. IL, United States) and CD4 counts measured with CyFlow Counter (Sysmex Asia Pacific Pte. Ltd., Singapore). Discordant immune response (DIR) was defined as virological suppression to <150 copies/mL and with a CD4^+^ T cell count spike of <500 cells/μL after at least 12 months of HAART. Patients with DIR were further categorized as DIR350 (CD4^+^ T-cell count <350 cells/μL) and DIR500 (CD4^+^ T-cell count between 350 and <500 cells/μL). The exclusion criteria included patients not receiving ART treatment, refusing to complete the consent form, and having a VL above 200 copies/mL. Given the exploratory nature of the study, all eligible participants during the study period were consecutively enrolled (see Table 1).

**Table 1:** Patients characteristics.

Characteristics	Total (*n* = 40)	CD4 < 350 (*n* = 11)	CD4 = 350–500 (*n* = 11)	CD4 > 500 (*n* = 18)	*p*-value
Age, year	37 (28–44.5)	40 (38–47)	37 (28–47)	32 (27.5–40.3)	0.094
Sex, male, (%)	25 (62.5%)	7 (63.6%)	8 (72.7%)	10 (55.6%)	0.648
Baseline CD4 count (cells/μL)	314.5 (213.3–489)	183 (153–294)	324 (280–395)	457 (228–561)	0.005^**^
CD4 (cells/μL) post 12 months on TLD	463 (343.3–646)	240 (178–340)	415 (364–461)	654 (563–720)	<0.0001^****^
Treatment duration (months)	14 (12–57)	14 (12–24)	36 (12–96)	14 (12–63)	0.250

Data are reported in median (QR) of patients, unless otherwise indicated. ^**^p < 0.01 and ^****^p < 0.001.

Descriptive and ordinal regression analyses were performed, with suitable measurements of central trends and a *p*-value of 0.05 indicating the confirmation of a relationship between variables. Demographic parameters such as age and gender were collected from all individuals. Comparison of categorical variables was tested using the chi-square test, whereas continuous variables (e.g., age) were compared using the unpaired *t*-test. Factors that associated with CD4 recovery post-ART were evaluated by simple and adjusted linear logistic regression. The coefficient (*B*) and 95% confidence interval (CI) were estimated. The Saveetha Medical College and Hospital Ethics Committee approved the study, and written informed consent was obtained from all the patients who participated in this study.

## Results

One hundred and twelve patients (*n* = 112) were screened at Saveetha Medical College and Hospital ART Centre (SMCH ART Centre) in southern India, between June 2023 and Dec 2024. Among them, 40 subjects met the study criteria and 22 (55%) were found to fall under the definition of DIR.

Among the 22 patients with DIR, 11 (27.5%) had a CD4 spike of <350 cells/μL (DIR 350) and the remaining 11 (27.5%) had a CD4 spike between 350 to <500 cells/μL (DIR 500) from baseline, at 12 months post-HAART. The remaining 18 (45%) were responders with both viral suppression and immune reconstitution above 500 cells/μL (Figure 1).

To identify factors associated with CD4 recovery following ART, linear regression analyses were performed. Variables including age, sex, baseline (BL) CD4 count, and treatment duration were first assessed in univariate models against CD4 count at 12 months post-ART. Predictors that were statistically significant in univariate analysis were then included in multivariate models. Univariate analysis indicated that age, baseline CD4 count, and treatment duration were significantly associated with CD4 recovery, while sex was not. Due to the limited sample size, inclusion of all three predictors in a single multivariate model risked overfitting; therefore, separate models were constructed to evaluate their independent effects.

**Figure 2:**
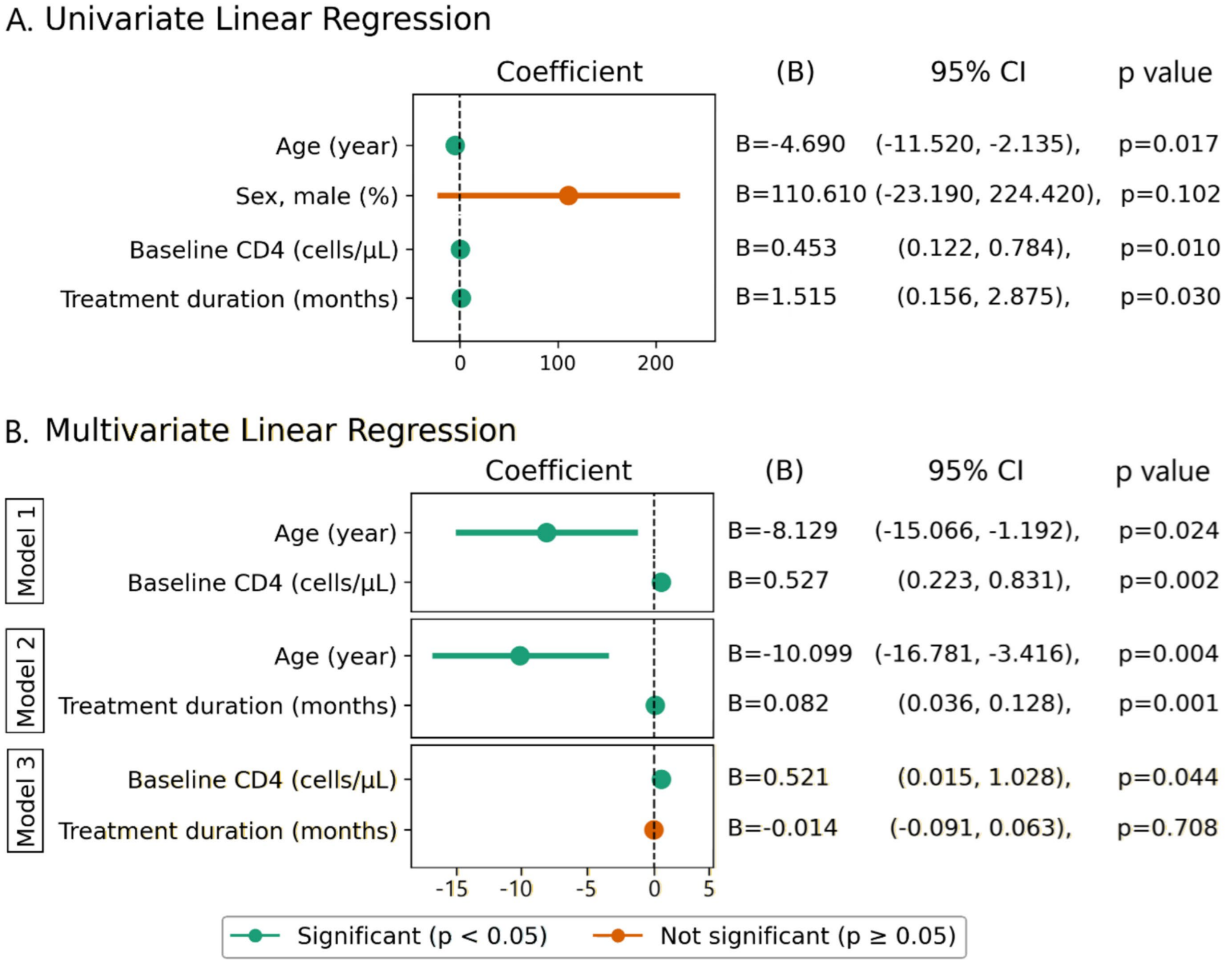
Factors associated with CD4 recovery post-ART. **(A)** Univariate linear regression analysis, **(B)** multivariate linear regression. Variables, for which *p*-values were less than 0.05 in univariate analysis, were considered as candidates. The candidate predictors were then included in a multivariate model, and variables, for which *p*-values were less than 0.05, were considered as independent predictors. *B*, coefficient; CI, confident interval.

In the model including age and BL CD4 counts, both variables were significant predictors. Higher baseline CD4 was strongly associated with higher current CD4 (*B* = 0.527, 95% CI: 0.223–0.831, *p* = 0.002), while older age was negatively associated (*B* = −8.129, 95% CI: −15.066 to −1.192, *p* = 0.024). In contrast, when baseline CD4 was excluded and age and treatment duration were analyzed together, both were significant: age showed a negative association (*B* = −10.099, *p* = 0.004), and longer treatment duration was positively associated with current CD4 (*B* = 0.082, 95% CI: 0.036–0.128, *p* = 0.001). These findings indicate that baseline CD4 and age are the strongest independent predictors of post-treatment CD4 count, while the effect of treatment duration is modest and appears confounded by baseline CD4 (Figure 2).

## Discussion

As per the National AIDS Control Organization (NACO) report of 2023, India has 2,544,000 PLWH, and of them, 1,690,000 are under HAART. Although the overall prevalence of HIV remains low (0.2%), challenges such as discordant immune response in this population may indirectly impede achievement of the 95:95:95 initiative by 2030. Patients with DIR are at increased risk of developing serious non-AIDS events (SNAEs) as well as AIDS-related events, and the management of these conditions may require additional prophylaxis (e.g., for opportunistic infections), frequent monitoring and may experience recurrent illness, leading to an increase in pill burden, health care visits and treatment fatigue, potentially compromising adherence to HAART and, consequently, viral suppression. Understanding the limitations to its success is crucial in enhancing individual responses to treatment and improving regimen durability. As per the WHO guidelines, HIV viral load monitoring is advised for routine identification of ART treatment failure, but CD4 cell counts for patients who are virologically suppressed remain an essential clinical and prognostic tool for identifying DIR. DIR, refers to the failure of HIV-infected individuals to achieve a satisfactory increase in CD4^+^ T cell counts despite successful suppression of viral replication with ART. This condition can lead to increased morbidity and mortality compared to individuals with optimal immune reconstitution.

Out of 40 patients enrolled in the study, 22 (55%) exhibited a discordant immune response with a CD4^+^ T-cell count below 500 cells/μL even after 12 months post-HAART and successful viral suppression. The average age of the patients with DIR was 38.5, and the study did not find any statistical correlation between age and the risk of developing DIR. While this finding is different from other studies that concluded the association of a one-year increase in age with a higher risk of developing DIR (28, 29), another study from Maharashtra, India, did not find any significant correlation between age and DIR, suggesting that factors other than age, such as comorbidities, treatment regimens (30) and major immune mediated factors like persistent immune activation and chronic inflammation, impaired thymic output, lymphoid tissue fibrosis, T-cell exhaustion and senescence, and ongoing immune dysregulation driven by co-infections, may play a more critical role in determining discordant immune response (7, 31, 32). There was no statistically significant difference in the prevalence of DIR among males and females in this study, unlike other studies, where male gender has been significantly found to be associated with a higher prevalence of DIR compared to females (33–36).

This cross-sectional study reports a higher prevalence of DIR (55%) compared to other studies in India that have reported a prevalence ranging from 5.6 to 10.02% (11, 28, 30). The increased prevalence rate in our study may be due to the difference in the CD4^+^ T-cell count cutoff value, which defines immunologic nonresponse, and also due to the small sample size compared to others. A global prevalence of DIR ranges from 7.6 to 41% and the definition for DIR used by most of these studies was a failure to increase in CD4 cell counts by 50–100 cells/μL after an year of HAART while the other studies considered the achievement of a predefined CD4 value, with thresholds that ranged from 200 to less than 500 cells/μL (16–19, 37–42). Among the studies done globally, Kelley et al. (42) report a higher prevalence of 41% and the definition used for DIR was a CD4^+^ T-cell count spike of <500 cells/μL from baseline, similar to our study and a PVL threshold of <1,000 copies/mL at 12 months of HAART. When the patients with DIR were further stratified as those with CD4 T cell spike of <350 cells/μL at 12 months post HAART, the prevalence was found to be 27.5%, a finding consistent with reports from other studies globally (37–39).

A low baseline CD4^+^ T-cell count was one of the key risk factors associated with immune discordance in our study (*p* < 0.05), which is consistent with many other studies reported in India (11, 28, 30) and globally (19, 37–41). Patients with baseline CD4 counts of <350 cells/μL were significantly associated with DIR at 12 months post HAART, like that observed in the EuroSIDA study by Florence et al.,2003. The association of a lower baseline CD4^+^ T-cell count with immunological non-response may be due to several factors, including impaired bone marrow hematopoietic function, decreased proliferative capacity, lower thymic output, dysfunction in certain cytokine expressions, and CD4^+^ T-cell destruction (5, 11, 33).

The best course of action for maximizing CD4^+^ T-cell recovery in DIRs remains unknown and the mechanism of immune reconstitution in this population remains unclear. As HIV in itself is a complex disease condition that includes chronic immune activation and ongoing inflammation despite successful treatment, the proportional contribution of mechanisms like immunological exhaustion, increased cell death, and fast T-cell turnover differs significantly from person to person, and hence several attempts by researchers to raise the level of immune reconstitution in immunological non-responders have not been successful. Hence, the pathophysiology exhibited in HIV pays the way for a customized treatment (5).

Many studies, including intensification with maraviroc or raltegravir, immunomodulatory drugs like chloroquine and its analogue hydroxychloroquine, statins, aspirin, anti-inflammatory cytokines and probiotics and prebiotics in addition to a standard ART regimen, have been evaluated for reducing immune reconstitution and activation, but not with much success (27, 43–46). The main reasons being adverse effects and the preexisting immune system abnormalities in HIV infection that seem to overpower the immunomodulatory effects of majority of candidates, thus necessitating a need for an adequate dose for an extended period of candidate interventions, in order to moderate low-level HIV replication, microbial translocation across damaged mucosal surfaces, and chronic coinfections which may contribute to persistent inflammation during influential virologic ART and finally reducing persistent immune. Hence, a safe, combined, adequate, and long adjunct therapy is the need of the hour.

The limitations of the study include its cross-sectional design, which only collects data on all variables at a single point in time, potentially missing information on adherence and concomitant infections due to changes in immunity over time. Further, the CD4/CD8 ratio was not available to evaluate its effect on immune recovery, as only CD4^+^ T cells testing was done as per WHO guidelines to monitor PLWH on ART. Other parameters like markers of immune activation and senescence /exhaustion and thymic dysfunction were not done and hence its association with the development of DIR is not assessed in this population. Secondly, the limited sample size would have affected the statistical power of the results. Thirdly, the impact of unidentified confounders would have affected the current study’s findings because of the small number of covariates we had to assess, and finally, the results might not be generalizable to larger groups because the data were gathered from a single institution, but these results do, however, mirror what we usually see in our clinical environment.

In conclusion, this study from an ART Centre in Chennai reports a DIR prevalence of 55% that was significantly associated with lower baseline CD4 of <350 cells/μL. The prevalence of DIR in this study is higher compared to other studies conducted in India, which could be attributed to the low sample size and differences in CD4^+^ T-cell count thresholds for defining DIR. Despite its small sample size, this study highlights the need for dedicated guidelines to manage patients with DIR despite virologically successful HAART, which would be supportive for achieving the 95:95:95 targets by 2030. As existing studies on DIR in India are largely retrospective, well-designed prospective studies are required to determine the incidence of immunological discordance and to assess its relationship with adherence, morbidity, mortality, demographic factors, and markers of immune activation and inflammation. Findings from such studies would inform policymakers and clinicians in developing evidence-based management strategies for this population.

## Data Availability

The original contributions presented in the study are included in the article/supplementary material, further inquiries can be directed to the corresponding authors.
